# DEMOGRAPHIC DIFFERENCES IN THE LONGITUDINAL EFFECTS OF SYMPTOMS ON FALLS AND FALL-RELATED OUTCOMES

**DOI:** 10.1093/geroni/igac059.1393

**Published:** 2022-12-20

**Authors:** Wenting Peng, Christina Miyawaki, Cen Mo, Yuqian Luo, Siyuan Tang, Minhui Liu

**Affiliations:** Central South University, Changsha, Hunan, China (People's Republic); University of Houston, Houston, Texas, United States; Central South University, Changsha, Hunan, China (People's Republic); Central South University, Changsha, Hunan, China (People's Republic); Central South University, Changsha, Hunan, China (People's Republic); Central South University, Changsha, Hunan, China (People's Republic)

## Abstract

Research has shown an association between symptoms and falls and fall-related outcomes among older adults. However, this association was primarily drawn from cross-sectional studies or studies with a single symptom. Using the 2011-2018 waves of the National Health and Aging Trends Study, we examined whether 1) prior-wave common co-occurring symptoms predicted later-wave fall-related outcomes and 2) demographics moderated the longitudinal effects of symptoms on fall-related outcomes among community-dwelling older adults. Falls and fall-related outcomes were self-reported falls, multiple falls, fear of falling (FOF), and FOF limiting activity. The number of symptoms (from 0 to 6) was calculated based on the presence of pain, insomnia, breathing difficulty, depressive symptoms, anxiety, and fatigue. Binomial logistic regression was used for data analyses. Our sample consisted of 9,060 participants who contributed 34,327 observations. These observations were aged between 65 and 79 years old (57.7%), female (58.4%), and non-Hispanic White (70.5%). Each additional symptom was associated with an increased risk of falls (Adjusted Odds Ratio [AOR]: 1.13, 95% CI: 1.11-1.15), multiple falls (AOR: 1.15, 95% CI: 1.12-1.18), FOF (AOR: 1.21, 95% CI: 1.18-1.24) and FOF limiting activity (AOR: 1.25, 95% CI: 1.21-1.29). Age, race/ethnicity, education, and living arrangement significantly moderated the relationships between symptoms and falls and fall-related outcomes. However, gender did not moderate the effects of symptoms on any outcomes. These findings suggest that symptoms longitudinally predict falls and fall-related outcomes. Common symptoms assessment and individual demographics should be incorporated into fall risk assessments and interventions.

